# Next Generation Sequencing identifies mutations in GNPTG gene as a cause of familial form of scleroderma-like disease

**DOI:** 10.1186/s12969-017-0200-2

**Published:** 2017-09-26

**Authors:** Abdelali Zrhidri, Saadia Amasdl, Jaber Lyahyai, Hanane Elouardi, Bouchra Chkirate, Laure Raymond, Grégory Egéa, Mohamed Taoudi, Said El Mouatassim, Abdelaziz Sefiani

**Affiliations:** 1 Centre de Génomique Humaine, Faculté de Médecine et de Pharmacie, Mohammed V University, Rabat, Morocco; 2 Département de Génétique Médicale, Institut National d’Hygiène, Rabat, Morocco; 3 Département de pédiatrie médicale, Hôpital d’Enfant, Rabat, Morocco; 4 Département de Génétique Moléculaire, Laboratoire Biomnis, Lyon, France

**Keywords:** Mucolipidosis III gamma, *GNPTG*, Whole exome sequencing

## Abstract

**Background:**

Scleroderma is a multisystem disease, characterized by fibrosis of skin and internal organs, immune dysregulation, and vasculopathy. The etiology of the disease remains unknown, but it is likely multifactorial. However, the genetic basis for this condition is defined by multiple genes that have only modest effect on disease susceptibility.

**Methods:**

Three Moroccan siblings, born from non-consanguineous Moroccan healthy parents were referred for genetic evaluation of familial scleroderma. Whole Exome Sequencing was performed in the proband and his parents, in addition to Sanger sequencing that was carried out to confirm the results obtained.

**Results:**

Mutation analysis showed two compound heterozygous mutations c.196C>T in exon 4 and c.635_636delTT in exon 9 of *GNPTG* gene. Sanger sequencing confirmed these mutations in the affected patient and demonstrated that their parents are heterozygous carriers.

**Conclusion:**

Our findings expand the mutation spectrum of the *GNPTG* gene and extend the knowledge of the phenotype–genotype correlation of Mucolipidosis Type III gamma. This report also highlights the diagnostic utility of Next Generation Sequencing particularly when the clinical presentation did not point to specific genes.

## Background

Scleroderma is a complex disease characterized by vascular involvement and progressive sclerosis of the skin and other organs. Its etiology is still unknown but it is likely multifactorial. The importance of the genetic contribution in this disease is still not completely understood. However, numerous papers were raised in this issue. Thus an increased chromosomal breakage was described with systemic scleroderma and segregated as a dominant marker [1]. Also, several genome-wide association studies disclosed multiple loci linked to increased risk of scleroderma [2].

In recent years, the use of massive parallel sequencing, or Next Generation Sequencing (NGS), is revolutionizing genetic investigation as well as clinical practice, mostly when dealing with rare diseases. The low cost of sequencing and growing bioinformatic capacity will permit the routine clinical use of these tools in the rapid diagnosis of Mendelian disorders with heterogeneous phenotypes. NGS can be used in several ways: as a panel targeting selected genes, as a test sequencing the coding regions of the genome (Whole Exome Sequencing/WES), or as an exam where the whole genome, with its coding and non-coding regions, is sequenced. This technology is also rapidly expanding into the genetic diagnostics field, expected to soon replace the standard Sanger sequencing. Recently, this approach allowed identification of novel gene variants and pathways that may contribute to scleroderma risk and/or severity [3].

Here we describe three Moroccan sibling presenting a familial form of scleroderma-like, and we highlight the crucial performance of WES in diagnosis issues.

## Methods

### Clinical studies

**Fig. 1:**
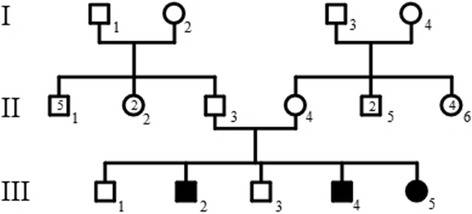
Family pedigree

The proband (III-2) is a Moroccan 15 years old boy, non-consanguineous, second child of five siblings among which the two youngest are affected (Fig. 1), and was initially admitted for genetic evaluation of familial scleroderma. His parents and older brother appear to be healthy suggesting an autosomal recessive inheritance pattern. Clinical examination, skeletal survey, Scl 70 antibody, and Skin biopsy, Electroencephalogram test, and muscle biopsy were carried out in the affected siblings.

### Whole Exome Sequencing

Whole Exome Sequencing (WES) was carried out in the proband (III-2) and his parents (II-3, II-4). In addition, Sanger sequencing was performed both in the parents and in the three affected siblings to confirm the results obtained. Genomic DNA was extracted from peripheral blood samples using the salting-out method. 500 ng of fragmented DNA (enzymatic fragmentation, Kapa Hyper Plus Kit) was amplified in compliance with user guide, and was subjected to enrichment with SeqCap EZ Human Exome v3.0 (Roche Nimblegen). The 64 enriched megabases were sequenced using an Illumina HiSeq 2500 system in rapid run paired-end mode (2x100bp). Raw data (bcl files) were converted in fastq files using bcl2fastq v1.8.4 (Illumina).

### Bioinformatics analysis

The sequence reads were aligned with the human genome reference sequence (University of California Santa Cruz, human genome assembly 19 (UCSC hg19)) recommended by GATK best practices: mapping was performed using BWA-MEM, variant calling using GATK (haplotypecaller). Annotation and filtering steps were performed using VariantStudio (Illumina). We first removed non exonic variants and variants indicated as synonymous, Variants files of parents and index case were confronted: only variants that fulfilled recessive inheritance pattern were selected. Among them, variants with allele frequencies above 1% in ESP6500 exome project. All variants predicted to be benign were removed from the list of variants. Amino acid conservation across different species was studies using the UCSC Genome Browser.

### Candidate gene sequencing

The putative mutation were validated via Sanger, sequencing the DNA of 5 family members with the following primers: F_ex4: 5′-GAAAACTGGTCCTTTGAAAACAG-3′; R_ex4: 5′-CCTGCACTCACGTGGACTC-3′ primer pair in exon 4 and F_ex8: 5′-CTGCATCCTCCACCTTCAG-3′; R_ex8: 5′-ACCTTCCTGCAGTTTTCCAG-3 primer pair in exon 9 of *GNPTG* gene. Sequencing was performed using BigDye Terminator v3.1 cycle sequencing, and the obtained sequences were analyzed on an ABI 3130 DNA Analyzer (Applied Biosystems).

## Results

### Patients

**Fig. 2:**
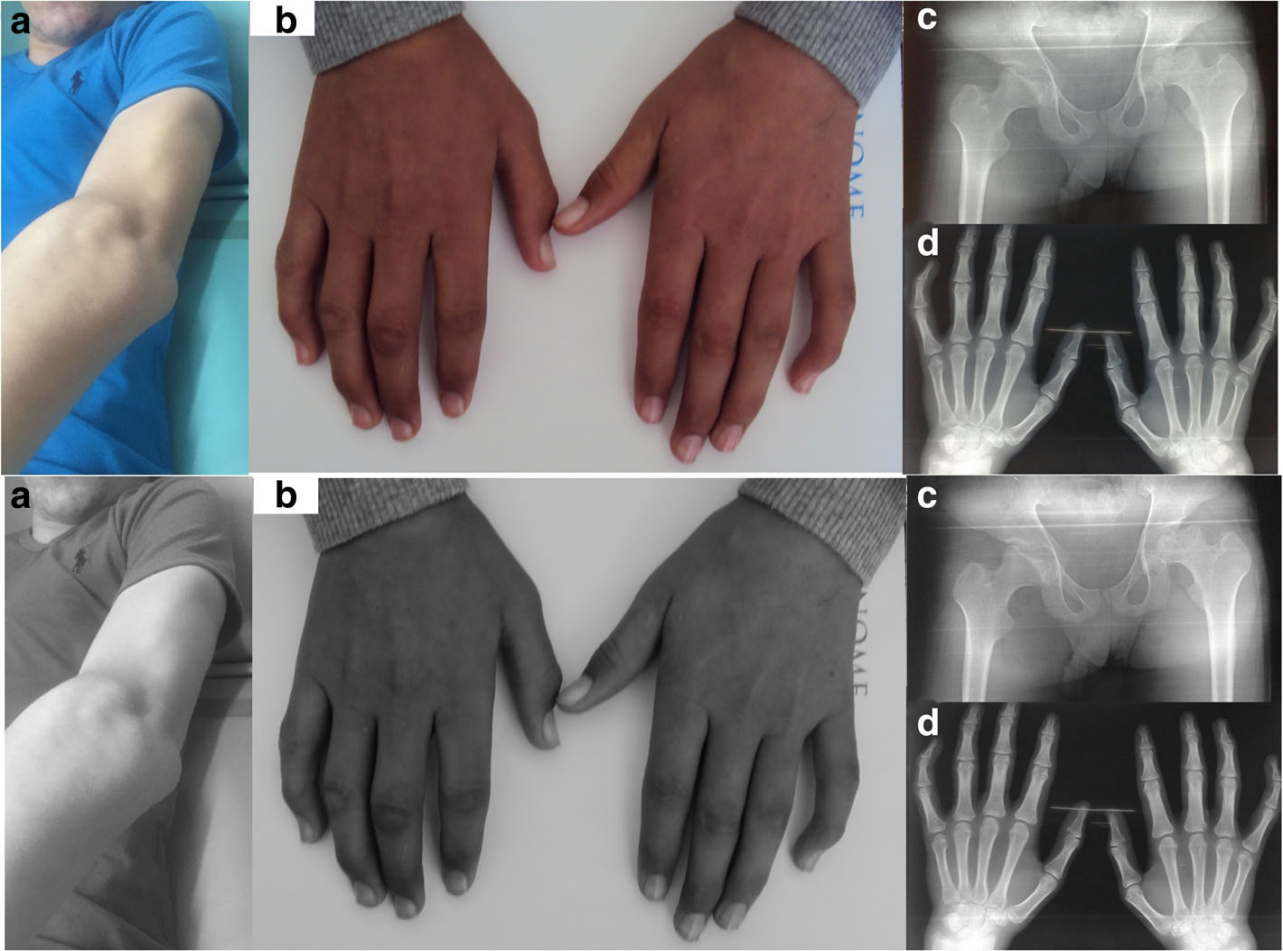
Clinical features of Scleroderma-Like. **a** Elbows flessum. **b** Joint stiffness of the fingers. **c** Osteonecrosis of the femur. **d** Bone demineralization with joint space narrowing over the hand

The patient (III-2), a 15 years male old, was the second child of healthy non consanguineous parents, born at term after an uneventful gestation and normal delivery. The perinatal period and first eightr years of life were uncomplicated. Physical and psychomotor developments were normal for age. First symptoms have appeared by the age of nine, with gradual evolution. Restricted joint mobility of the fingers, wrist and toes was observed in addition to dry and tightening of the skin. There was no history of preceding trauma or illness. Large joints were gradually involved with limited mobility of arms, knees and hip joints as well as spinal column. Physical examination was notable for retracted, hard, tight and dry skin over the hands, abdomen and lower limbs as well. Additional findings included elbows flexum (Fig. 2(a)), tendon retraction of the wrist, metacarpophalangeal (MCP) and proximal interphalangeal (PIP) joints (Fig. 2(b)), as well as limitation of hip abduction and rotation. Raynaud syndrome, hyper- and hypopigmentation areas; Nevertheless, There was no evidence for other features of scleroderma namely telangiectasia, erythema, edema, violaceous color, waxy appearance, and warmth. The remaining finding showed limping gait, genu valgum, kyphosis and absence seizures. Further evaluation revealed an inflammatory syndrome with elevated ESR and CRP levels estimated at 32 mg/l and 41 mm/h respectively, anti Scl 70 antibodies were positive, while Anti-nuclear and anti-DNA antibodies were negative. Skin biopsy displayed thinned epidermis, fibrous dermis with rare vessels, rarefied and atrophied adnexal structures, fibrous hypodermis. X-ray examinations disclosed bilateral osteonecrosis of the femur (Fig. 2(c)), bone demineralization with joint space narrowing over the hand (Fig. 2(d)). Electroencephalogram showed slow waves with slow spikes located in the anterior temporal region. Whereas, echocardiogram, muscle biopsy were normal. Family history revealed a young brother (III-4) and sister (III-5), 12 and 9 years old respectively, who exhibited almost the same clinical disease progress. They had first symptoms between the ages of 5 and 8 years, with progressive skin retraction. Clinical examination revealed retracted, hard, tight and dry skin over the hands, and lower limbs as well, slight limp, tendon retraction of the wrists, MCP and PIP joints, flexum of the two elbows. Paraclinical investigations showed inflammatory biological syndrome, anti Scl 70 antibodies, anti-nuclear and anti-DNA antibodies were negative, bone demineralization with articular pinching, skin biopsy was compatible with scleroderma. This was interpreted as familial form of scleroderma genetic-like. The three sibling were placed under systemic corticotherapy and physiotherapy. Subsequent evaluation revealed stabilization of cutaneous lesions and normalization of the inflammatory parameters.

### Molecular genetic and bioinformatic analysis

**Fig. 3:**
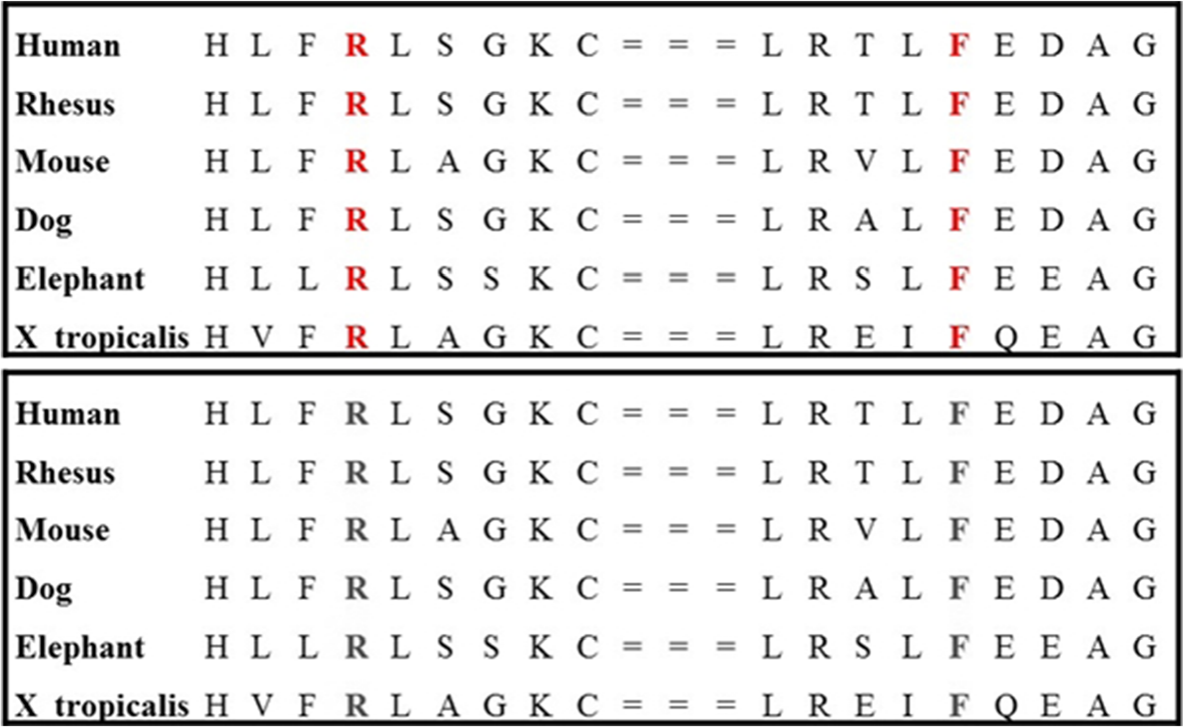
Protein multiple sequence alignments (PMSA) of the corresponding Mutations p.Arg66Ter and p.Phe213Ter. Arg (R) and Phe (F) are highly conserved in different species

A total of 52,686 variants in 13,765 genes were detected. Following the step-by-step filtering protocol described above, we detected a compound heterozygous mutation c.196C>T (p.Arg66Ter) in exon 4 and c.635_636delTT (p.Phe213Ter) in exon 9 of the *GNPTG *gene (NM_032520), resulting in premature stop codons at amino acid 66 and 213. As shown in Fig. 3, these variants affects highly conserved residues.

Sanger sequencing confirmed these mutations in compound heterozygous state in the affected subjects (III-4, III-5). The parents were heterozygous carriers. The father carries the mutation c.635_636delTT and the mother carries the mutation c.196C>T, showing cosegregation of these mutations with the disease phenotype.

### Enzymatic analysis

The pathogenicity of the variant in *GNPTG* was confirmed by assessing the serum enzyme activities of hexosaminidase, α-N-acetylglucosaminidase, β-mannosidase and β-glucuronidase, which are lysosomal enzymes known to be dependent on mannose- 6-phosphate (M6P) targeting. Lysosomal enzyme activities were found to be obviously increased in the serum of proband compared to levels in healthy controls, thus confirming the diagnosis of Mucolipidosis type III gamma (Table 1).

**Table 1:** Activity of acid hydrolases in our patient compared to normal values

	Activities in affected patients	Range of normal values
Hexosaminidase	3434 nkat/L	148–337 nkat/L
Hexosaminidase A	207 nkat/L	24–44 nkat/L
α-L-Fucosidase	1195 nkat/L	10–170nkat/L
β-D-Glucuronidase	1416 nkat/L	15–93 nkat/L
β-Mannosidase	975 nkat/L	50–210 nkat/L

## Discussion

Mucolipidosis type III (ML III, MIM 252605), known as pseudo-Hurler polydystrophy, is an autosomal recessive disease caused by deficiency of UDP-N-acetylglucosamine 1-phosphotransferase [4]. This enzyme is requested during the labeling of the acid hydrolases (AH) by the mannose-6-phosphate (M6P) in the cis Golgi cisterns [5]. Only tagged AH are binded to M6P-receptors in the trans-Golgi and then delivered to the lysosome.The lack of M6P result in impaired targeting of hydrolases to lysosome, leading to their excessive release into the extracellular compartment [6]. As a consequence, cells accumulate undigested macromolecules in their endosomes/lysosomes [7]. Clinically, ML III is milder than other forms of mucolipidosis; this begins often from 3 years with slow coarsening of the facial features, growth deficiency, progressive joint stiffness, claw hand deformity, gait impairment with hip disease, scoliosis, dysostosis multiplex, normal intelligence or mild intellectual disability [8, 9]. Laboratory diagnosis is readily made by demonstrating increased lysosomal enzyme levels in serum, and reduced levels in extracts of cultured fibroblasts [10]. Clinical presentation of the ML III is heterogeneous [11], and constitutes a challenge area with different diagnosis. Thereby, ML III α/β and ML III γ are almost indiscernible Clinically [12]. Rheumatologic disorders, such as juvenile idiopathic rheumatoid arthritis, progressive pseudorheumatoid arthritis of childhood and scleroderma, are usually suspected in individuals with ML III [13, 14]. Our proband exhibited notably dermatological and rheumatological manifestations mimicking scleroderma. He had retracted, hard, tight and dry skin, Raynaud’s syndrome, hyper- and hypopigmentation areas. However, these alterations were seldom noted in individuals with ML III [15]. Skin biopsy in these patients revealed increased cytoplasmic lysosomal granules or inclusions in lymphocytes and fibroblasts in various tissues. This histopathologic finding is known as I-cell phenotype [16], and was not obvious in our family. In addition, our patient had rheumatological manifestations mainly joint stiffness, elbows flexum, tendon retraction (wrist, MCP and PIP joints), limitation of hip abduction/rotation, limping gait, genu valgum, and kyphosis. Other finding were bilateral osteonecrosis of the femur, bone demineralization and joint space narrowing over the hand. Joint manifestations have been described in 46% to 97% of patients with scleroderma [17], predominantly at the hand joints, in particular the MCP and PIP joints [18, 19]. Radiological signs were mostly joint space narrowing, bone demineralisation, acro-osteolysis, flexion contracture, and calcinosis [18]. However, bone necrosis has been rarely associated with scleroderma [20]; This was considered as a common complication of steroid therapy, and microvascular involvement as well [21, 22].

In this paper, due to clinical heterogeneous presentation of the disorder as well as recurrence in sibling, we opted for the WES which allowed to maintain the right diagnosis and exclude other differential ones. By that, two compound heterozygous mutations causing ML III gamma in Moroccan family were identified in the *GNPTG* gene, including a maternally inherited nonsense mutation c.196C>T (p.Arg66Ter), and a paternally inherited deletion mutation c.635_636delTT (p.Phe213Ter), in all three patients. The first variant is a single nucleotide substitution located in exon 4, described once as a pathogenic mutation that is predicted to severely damage the protein product [23]. The second mutation is a small deletion c.635_636delTT (p.Phe213Ter) located in exon 9, this variant was reported in heterozygous state in healthy people, but never seen in affected individuals, making it a new pathogenic mutation related to mucolipidosis. Based on UCSC server (Fig. 3), these mutations lies within a conserved domain. To the best of our knowledge, 24 different mutations in the *GNPTG* gene have been reported, including six missense/nonsense mutations, seven small deletions, four small insertions, two gross deletions and five splice site mutations [http://www.hgmd.cf.ac.uk/ac/gene.php?gene=GNPTG]. However, no *GNPTG* mutations have been reported in the Moroccan population yet.

## Conclusion

We describe here a Moroccan family with ML III mimicking scleroderma, thus we highlight the crucial interest of WES to straighten the suitable diagnosis among several suspicions. We see herein how the analysis of three individuals from a single family helped to narrow the list of candidate mutations, and delineate the disease gene using a standard clinical assay.
